# Response Identification in the Extremely Low Frequency Region of an Electret Condenser Microphone

**DOI:** 10.3390/s110100623

**Published:** 2011-01-10

**Authors:** Yih-Nen Jeng, Tzung-Ming Yang, Shang-Yin Lee

**Affiliations:** Department of Aeronautics and Astronautics, National Cheng Kung University, Tainan, 70701, Taiwan; E-Mails: p4893120@mail.ncku.edu.tw (T.M.Y.); p46954371@mail.ncku.edu.tw (S.Y.L.)

**Keywords:** response identification, extremely low frequency, electret condenser microphone, time frequency analysis

## Abstract

This study shows that a small electret condenser microphone connected to a notebook or a personal computer (PC) has a prominent response in the extremely low frequency region in a specific environment. It confines most acoustic waves within a tiny air cell as follows. The air cell is constructed by drilling a small hole in a digital versatile disk (DVD) plate. A small speaker and an electret condenser microphone are attached to the two sides of the hole. Thus, the acoustic energy emitted by the speaker and reaching the microphone is strong enough to actuate the diaphragm of the latter. The experiments showed that, once small air leakages are allowed on the margin of the speaker, the microphone captured the signal in the range of 0.5 to 20 Hz. Moreover, by removing the plastic cover of the microphone and attaching the microphone head to the vibration surface, the low frequency signal can be effectively captured too. Two examples are included to show the convenience of applying the microphone to pick up the low frequency vibration information of practical systems.

## 1. Introduction

Because of the rapid development of web sites and computer software and hardware, peripheral devices from electret condenser microphones [1–3] to webcams to printers are becoming more and more popular. Since they are all produced on a mass scale, they are often very cheap yet still of high quality. Thus, there is potential to develop a notebook or PC-based data acquisition system using a small electret condenser microphone as a sensor [4–7]. Although such systems are quite cheap, they can be used in certain higher-end applications. For example, by properly equipping one with suitable software, it can pick up dominant vibration information from a system in operation to serve as a type of non-contact warning equipment. In [8], we applied it to pick up the arterial pulse pressure information of human wrist vessels, suggesting the potential for constructing a diagnosis system. Moreover, it would be possible to promote them to achieve high quality products if more and more people researched and used them. The authors believe these systems will enhance the research potential of under-developed countries as well as undergraduates and high school students in most countries. Such efforts, with reasonable investment, will also benefit developing health care systems and environmental monitoring systems.

It is well known that acoustic signals may involve information related to speech, structure vibration, fluid motion, and wave propagation, *etc.* They frequently contain much information transferred in direct and indirect manners through solids, liquids and gases. Thus, an acoustic data string may include the operating conditions of one or more systems and their components [4–10]. Specifically, one can easily pick up these acoustic signals by putting the microphone at any place near the measured system. The only question is whether the microphone can capture the signals of interest when they are minor wave modes of the collected data string.

The frequency response [11–13] of an electret condenser microphone is usually in the range of 20 Hz–20 kHz. This can be easily verified by using a commercial search engine such as Google. This range reflects the fact acoustic waves are propagated in open space. In other words, extremely low frequency information cannot be easily collected when the microphone is arbitrarily placed anywhere. This study aimed to show that, in a specific environment, the lower limit of the frequency response range of a microphone can be as small as 0.5 Hz.

Since acoustic data may contain information emitted by multiple sources, it is often very complicated and therefore difficult to extract the embedded information [4–10]. Generally, a continuous time series data may involve a trend, periodic part and noise. Instrumental data are often contaminated by a mean trend resulting from processes other than those of primary interest [14]. For example, if a data acquisition system is not properly adjusted, the selected data might include a drift and/or trend during an experiment. In [15], it was pointed out that in many practical situations, processing experimental observations of superposed phenomena having different time scales is required. In other words, a trend is frequently found because it can be defined as those frequency components whose periods are longer than the records [16]. Before applying the spectral method, this embedded trend should be removed so the resulting spectrum is not seriously contaminated [14,17].

To examine the periodic part, the Fourier method is successfully applied to those problems whose wave components only involve fixed amplitudes and frequencies [18–24]. There are two well known time frequency transforms, named the Gabor and Morlet transforms [19–24]. These transformations are suitable for resolving the time dependent amplitude and frequency. In this study, the iterative Gaussian filter [9,10,25] and modified Gabor transform [9,10] are used to remove the trend and perform time frequency analysis, respectively.

This paper is organized as follows. Section 2 presents the experiment setup and theory. Section 3 then briefly discusses the related data analysis tools. The performance of the proposed method is shown in the Results and Discussion in Section 4. Finally, the paper is concluded in Section 5.

## 2. Experimental Setup and Theory

**Figure 1.**
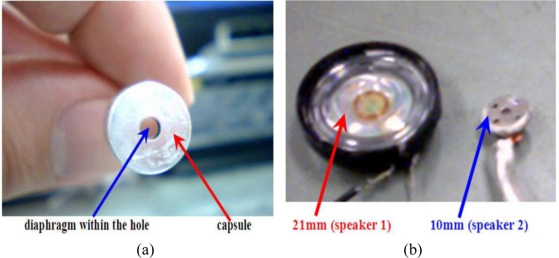

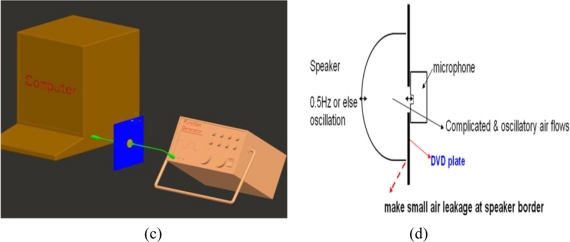
**(a)** Schematic diagram of an electret condenser microphone without the plastic package. **(b)** General view of two speakers; 21 mm in diameter (left) and 10 mm in diameter (right). **(c)** Schematic diagram of the overall arrangement. **(d)** Schematic diagram of the DVD plate, microphone and speaker.

An electret condenser microphone has a diaphragm and a back plate serving as positive and negative electrodes, respectively. These two plates are separated by a thin spacer so they become an electrical capacity. An integrated circuit involving the field effect transistor will amplify the signal induced by the capacity’s change. These facilities and PC board are enclosed by a capsule. The front head of the capsule has a small circular hole where the diaphragm stands at the other end. All acoustic or sound waves propagating into the hole will reach the diaphragm and cause it to vibrate. At that moment, the distance between the diaphragm and back plate is changed accordingly. Thus, the electrical capacity between the two poles changes so a time dependent signal is produced. Since the output voltage is proportional to the first order time derivative of the capacity, the system output is linearly related to the first order derivative of the acoustic pressure. In general, for an electret condenser microphone used in a notebook or PC, the hole in the front head of the capsule is very small. For example, the hole of the microphone shown in Figure 1(a) has a depth of 0.2 mm and a diameter of about 2 mm.

When the microphone is exposed to air to receive a sound wave, a small amount of acoustic energy reaches the diaphragm whose threshold sound pressure level is about 40 dB. Most electret condenser microphones cannot faithfully capture the signal when the frequency of the acoustic wave is lower than 20 Hz. This is because when the corresponding sound pressure level is lower than 40 dB, the acoustic wave cannot provide enough force to actuate the microphone’s diaphragm. Based on this interpretation, it is interesting to see what happens when the acoustic wave’s amplitude is large enough. To properly answer this question, an experiment setup and procedure are designed as follows.

This study uses a commercial and light electret condenser microphone with specifications of 20 Hz to 20 kHz, 100 mw, 32 Ω, 105 dB and sound pressure level sensitivity of 1 kZ ± 2%. The other experimental facilities include a function generator (Iwatsu Inc. FG-350), two UNO speakers (10 mm and 21 mm in diameter [Figure 1(b)], and a sampling rate within a range of 20 Hz-20 kHz), and a digital audio board (Onkyo Inc. SE-150PCI, SN ratio 100 dB, 0.3–44 kHz, and sampling rate runs from 32 to 192 kHz).

The experiment and data analysis involve the following steps: first, a function generator sends a sine wave with a prescribed frequency (ranging from 0.5 to 10 Hz) to the speaker to emit acoustic waves. After propagating into the small air cell formed by the hole in the front end of the capsule, the small pressure wave’s signal is then reflected by the microphone’s voltage output. The analogue signal is then converted into a digital signal through a digital audio board. The digital data is saved in a PC using the Microsoft XP windows system. The corresponding schematic diagram is shown in Figure 1(c). Notably, this system can be easily updated using other function generators and computers. Moreover, the audio board may or may not be a built-in audio board of a PC or notebook.

The schematic diagram of Figure 1(d) shows the speaker and microphone are on two sides of a DVD plate (1.22 mm thick and with a small hole of 2 mm diameter). To ensure a fixed experimental environment, these three pieces are clamped together by a small vise. The small hole of a DVD plate, the front space of the speaker and the air cell containing the diaphragm of the microphone are then connected to one another to form a small air-chamber. Once an acoustic wave is generated by the speaker, instead of propagating into open space, most of its energy is confined within the chamber. Thus, a large fraction of the acoustic wave should reach the diaphragm of the microphone. Otherwise, if the DVD plate is not used and the microphone is just put near the speaker, only an infinitesimally small fraction of acoustic energy reaches the diaphragm.

Several test runs showed, if the DVD plate, microphone and speaker are tightly attached to one another without any air leakage, the microphone cannot receive any signal when the input frequency is less than 5Hz. The reason is the speaker’s power is too low to produce an effective volume change in the small airtight chamber. In this study, the region between the fringe of the speaker and DVD plate is partially filled with a thin layer (≤2 mm) of jelly (produced by Leo Lubricants PVT.LTD). On the other side of the plate, the microphone and plate are firmly bonded by glue. This setup produces several air channels on the fringe. Now air is allowed to flow-in and flow-out through the fringe of the speaker. This reduces the required threshold energy to effectively actuate the speaker so the microphone may receive a prominent signal. After a series of trial and error experiments, it was determined that clear signal output conditions are achieved if the speaker’s perimeter filled with the jelly is roughly less than 80 percent. According to the specifications of the manufacturer, it is reasonable to state that the sound pressure level of the incident acoustic wave is larger than 40 dB in this specific environment.

To diagnose the vibration information of a system of interest, the microphone is loosely attached to a suitable surface. Now the solid surface and the tiny air cell containing the diaphragm of the microphone form a small air chamber with small air leakage. As the oscillatory and relative motion between the solid surface and microphone presents, a pressure wave is induced and propagates into the enclosed air. The relative motion can be achieved once the microphone and solid surface are not firmly bound together. For example, one may hold the microphone by hand or use a tape to loosely bind the microphone and surface together. In the former case, a human hand kept the microphone from being synchronized with the surface. For the latter method, the output electric wire of the microphone provides the mechanism of non-synchronization between the microphone and the solid surface. Now the acoustic energy reaching the diaphragm is not infinitesimally small. When the sound speed is 330 m/s, all the signals whose frequencies are prominently less than 1.65 MHz (≈330 m/0.2 mm) are not the longitudinal standing waves in the normal direction of the diaphragm. In other words, in such a specific environment, the microphone certainly has the potential of also capturing acoustic waves in the 0.5–20 kHz range.

## 3. Tool of Data Analysis

Because the data picked up by the microphone system may involve drift and/or trend, it frequently involves a monotonic non-periodic part. Therefore, a time series data string, *y_j_* = *y*(*t_j_*), *j* = 0,1,2...,*J*, can be written in the following form [9,10]:

(1) $$y_{j}=\sum_{l=0}^{J-1}\left[b_{l}\;\text{cos}\;\frac{2\pi t_{j}}{\lambda_{l}}+c_{l}\;\text{sin}\;\frac{2\pi t_{j}}{\lambda_{l}}\right]+\sum_{n=0}^{N}a_{n}t_{j}^{n},\;\;\;\;0\;<\;j\;<\;J$$

where *t_j+1_* − *t_j_* = Δ*t* = constant, *l* is the mode index, and *λ_l_* = *J*Δ*t* /*l* is the wavelength of the *l*-th mode, the second summation represents the non-sinusoidal drift and/or trend and *N* represents the largest power for which *a_n_* → 0 for all *n* > *N*. For most engineering applications, *N* = 250 is a reasonable value. The non-sinusoidal trend is interpreted as the sum of monotonic parts and all the Fourier modes whose wavelengths are longer than the data span *T* = *J*Δ*t*. This study uses the iterative Gaussian smoothing method in the spectral domain to serve as a high-passed filter. It can be proven the resulting response takes the following form [9,10,25]:

(2) $$y_{m}^{'}=\sum_{l=0}^{J-1}{\left[1-a\left(\sigma /\lambda_{l}\right)\right]}^{m}\left[b_{l}\;\text{cos}\left(\frac{2\pi t}{\lambda_{l}}\right)+c_{l}\;\text{sin}\;\left(\frac{2\pi t}{\lambda_{l}}\right)\right]$$

where *y′_m_* represents high frequency response after applying the iterative Gaussian smoothing method for *m* cycles and *a*(*σ*/*λ_l_*) = 2*π*^2^*σ*^2^/*λ_l_*^2^ represents the attenuation factor, in which *σ* is the smoothing factor of the Gaussian smoothing method. In this study, the iteration parameter *m* and smoothing factor *σ* use the value of 127 and 0.8*T*, respectively. These parameters means that the filter’s transition zone is *λ_2_/λ_1_=2* and *λ_1_ ≈ σ/0.772 ≈ 1.036T*. More specifically, all wave modes of *y′_m_* with wavelength *λ ≤ 1.036T* are almost the same as that of original data and those wave modes with wavelength *λ ≤ 2.072T* are removed. Now the trend is ultimately removed. Because this result is derived by assuming the data span running from –∞ to ∞, small errors are thus induced by the missing data beyond the two ends. Next, the zero crossing points around the two ends can be located by a search procedure and interpolation formula. After dropping the data segments beyond the two zeros, a monotonic cubic interpolation [26] is used to redistribute the data into uniform spacing whose points equal an integer power of 2. Subsequently, the odd function mapping is used to double the data span. Finally, the Fast Fourier Transform (FFT) [18] will generate a Fourier sine spectrum. This spectrum reflects many details in the low frequency region because the trend has already been removed and all the required periodic conditions are ensured by the odd function mapping.

In this study, the following Gabor wavelet transform using the Gaussian window (with a given window width *a* on the time domain) is used for the sinusoidal data *y′_m_*:

(3) $$G(f,\tau )=\frac{1}{\sqrt{a}}\int_{0}^{T}y_{m}^{'}(t)e^{-2i\pi \;f(t-\tau )}e^{-{\left(t-\tau \right)}^{2}/\left(2a^{2}\right)}\mathrm{dt}$$

in which *τ* denotes the central time instant of the Gaussian window and *f* is the central frequency index on spectral domain. By scanning both *f* and *τ* over the desired range of time-frequency domain, the desired two-dimensional Gabor wavelet coefficient plot can be obtained. It can be proven it is about equal to the following form [9,10]:

(4) $$G(f,\tau )\;\approx \sqrt{\frac{a\pi }{2}}\sum_{l=1}^{J-1}\left\{\left[d_{l}-{\mathrm{ie}}_{l}\right]e^{i2{\pi f}_{l}\tau }\;\;\;\text{exp}\left[-2a^{2}\pi^{2}{\left(f_{l}-f\right)}^{2}\right]\right\}$$

where *d_l_* ≈ (1−2π^2^σ^2^/λ*_l_*^2^)*^m^ b_l_* and *e_l_* ≈ (1−2π^2^σ^2^/λ*_l_*^2^)*^m^ c_l_* are Fourier spectrum of *y′_m_*. These relations indicate the wavelet coefficient is just an inverse FFT of a finite spectrum band specified by an associated Gaussian window whose window width is 1/(2*aπ*) and is centered at the frequency of *f*. Note that the resulting wavelet coefficients are subject to the blur effect of the uncertainty principle [9,10,19–24].

## 4. Results and Discussion

The experimental setup of Figure 1 was used to examine the low frequency response of the microphone three times. Each experiment was done at a different time. To check the repeatability, the facilities of Figure 1(d) were re-assembled in every experiment in which the jelly was sprayed on about 70% to 80% fringe of speakers. The input frequency of the function generator was set by rotating the knob on the control panel. The following frequencies are given during each cycle of the experiment, say, approximately 0.5, 0.7, 0.8, 0.9, 1, 1.5, 2, 3, 4, 5, 6, 7, 8, 9, and 10 Hz, respectively. The knob of the input voltage was kept at the same level, say one voltage. Software (Goldwave) was used to convert the signal into the 16 bit single channel data with a sampling rate of 8 kHz. The recording period was 10 seconds.

**Figure 2.**
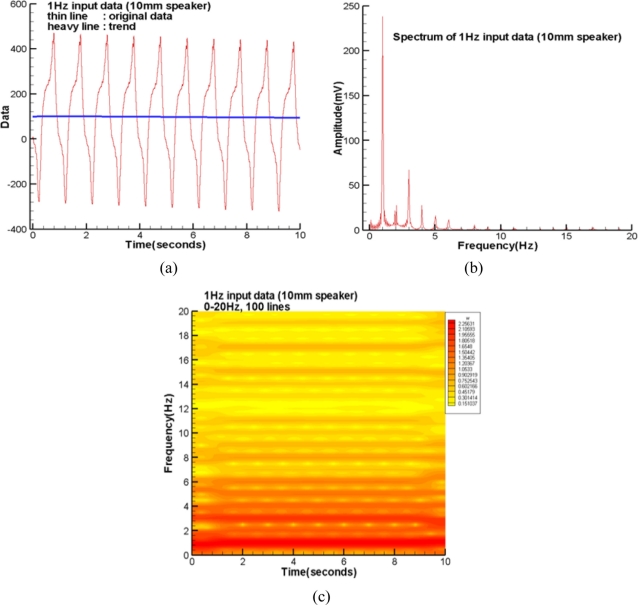
**(a)** Raw data and trend of the first cycle at 1 Hz (10 mm speaker). **(b)** Spectrum of the first cycle at 1 Hz (10 mm speaker). **(c)** Spectrogram of the first cycle at 1 Hz (10 mm speaker).

The thin solid line of Figure 2(a) plots the raw data of the first test cycle of the speaker (10 mm diameter) with an input sine wave of 1 Hz. The heavy solid line is the extracted smooth trend applying the iterative Gaussian filter in the spectral domain with *m* = 127 and σ = 5 seconds. The corresponding spectrum and spectrogram are plotted in Figure 2(b,c), respectively.

They tell us the 1 Hz mode is much larger than all the harmonics and noise. These minor extra-modes reflect the resonance within the air-cell and complex flow field induced by the oscillatory air stream through the air leakage channels. Notably, the split modes around regions of *t* = 0 and 10 seconds of Figure 2(c) are those errors introduced by the missing data beyond the two ends. If the region with jelly is smaller than the 50% fringe of the speaker, the air stream through the channel without jelly should induce a complex flow field within the air chamber [8]. Therefore, both the resulting spectrum and spectrogram become much more complicated than those shown in Figure 2(b,c), respectively. Fortunately, their dominant modes show similar characters. These results are not shown here because of the length limitation.

**Figure 3.**
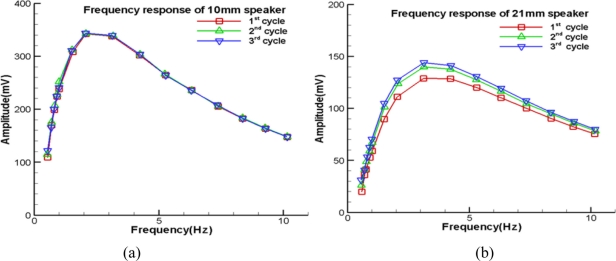
**(a)** Low frequency response of 10 mm speaker. **(b)** Low frequency response of 21 mm speaker.

Figure 3(a,b) shows the low frequency response of the corresponding input frequency of two speakers, with diameters of 10 mm and 21 mm, respectively. Seemingly, the scattering between three test cycles of Figure 3(b) is slightly larger than that of Figure 3(a). The differences between the three cycles of these figures are the experimental uncertainty of tuning the knobs of the input frequency and jelly spray region. Nevertheless, their relations between amplitude and frequency are similar to each other.

These figures indicate the existence of a threshold frequency, which is about 2.5 Hz for the 10 mm speaker and 3 Hz for the 21 mm speaker, respectively. Once the input frequency decreases and is smaller than the threshold value, the efficiency of the speaker and microphone decline rapidly. Thus, as shown in Figure 3, the corresponding response deteriorates significantly. On the other hand, as the input frequency increases beyond the threshold value, the frequency response reduces too. The reason is that, when the input frequency is larger than the threshold value, the pre-set air leakage may gradually become partially airtight. In fact, the air stream through the speaker’s fringe releases the mechanical resistance of the speaker’s output. As the speaker’s oscillatory frequency increases, the velocity of the induced air stream increases and therefore the viscous force increases as well. This viscous damping is the additional load of the speaker so its efficiency deteriorates. Despite these two deficiencies, Figure 3(a,b) shows the sine wave information of the function generator can be captured by the microphone in the low frequency region of 0.5–10 Hz. About the range of 10–20 Hz, if an incident acoustic wave has the same amplitude of one of the previously discussed sound waves, the corresponding sound pressure level is clearly larger than that in the range of 0.5–10 Hz. We can firmly expect it will be picked up by the microphone. Therefore, it is reasonable to extend the same conclusion to 0.5–20 Hz.

**Figure 4.**
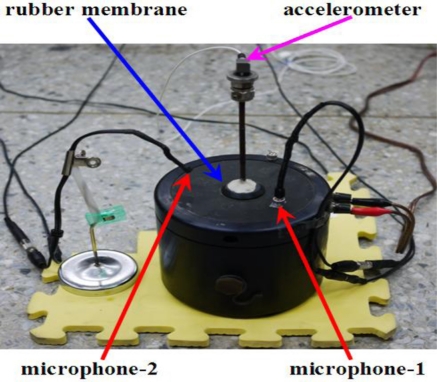
Schematic of the experimental setup of the exciter: the accelerometer is on top of the central bar; microphone-1 is attached to the membrane; and microphone-2 is at 10 cm above the membrane.

To further examine the microphone system together with the spectrum and spectrogram generators, the data of an electrodynamic Modal Exciter (B&K, Type 4824) is examined. The data is collected by an accelerometer with a sampling rate of 500 points/second. The accelerometer is ceramic, with a resolution of 100 mV/g for a 0.5 to 3 kHz signal. The A/D card (NI USB·6210) has a 16 bit resolution with a maximum sampling rate of 250 kbytes /second. The input signal to the exciter is given by an FG-350 function generator (IWATSU Electronic Co. Ltd) and monitored by an HP54603B oscilloscope. For convenience, the input signal is given in a sequence of stepwise frequencies which are roughly 3, 5, and 9.5 Hz, respectively. Figure 4 illustrates the experimental setup including the exciter, accelerometer, and two small electret condenser microphones. The accelerometer is located on top of the bar at the central part of the rubber membrane. One microphone is attached to the rubber membrane of the exciter and the other is 10 cm above the exciter.

**Figure 5.**
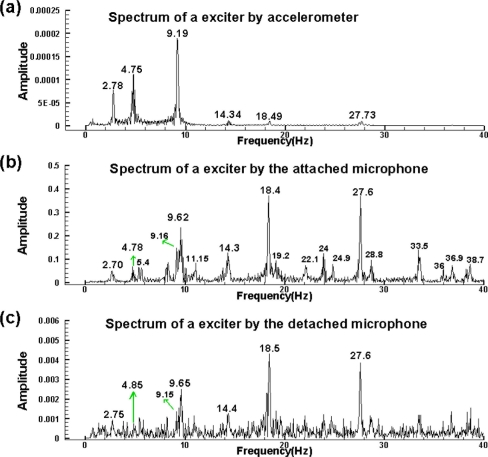
Spectra of three sensors: **(a)** the accelerometer. **(b)** microphone-1 attached to the membrane. **(c)** microphone-2 at 10 cm above the membrane.

The accelerometer measures the vertical acceleration of the central rubber membrane. On the other hand, the microphone’s output is proportional to the vertical velocity. To achieve a reasonable comparison, the accelerometer data is integrated once. The three resulting spectra after applying the above mentioned post processing are plotted as seen in Figure 5. The results of the accelerometer, attached and detached microphones are shown from top to bottom, respectively. The spectrum of the accelerometer is much smoother than the two microphone spectra because of the data integration. Seemingly, the most dominant modes of the attached microphone consist with that of the accelerometer. Further, the attached microphone also picks up many harmonics and modes combined from harmonics and sub-harmonics. These acoustic signals are too small to actuate the accelerometer. The dominant modes of detached microphone are the same as that of the attached one [see Figure 5(c)]. However, in the low frequency region less than 5 Hz, the detached microphone cannot prominently reflect the 2.7 and 4.8 Hz modes because the corresponding acoustic energies of these signals are small. Those additional minor modes of the detached one, which are excluded by the attached microphone, are information from the environment.

**Figure 6.**
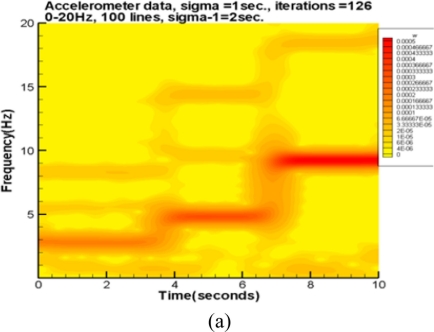

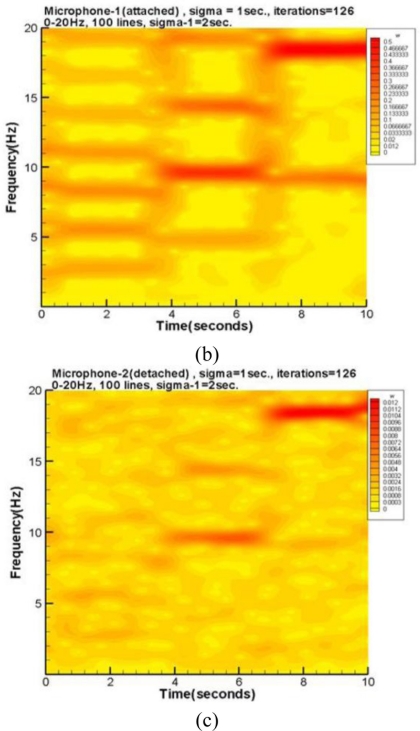
Spectrograms corresponding to those shown in Figure 5: **(a)** the accelerometer. **(b)** microphone-1 attached to the membrane. **(c)** microphone-2 at 10 cm above the membrane.

Figures 6(a–c) are spectrograms corresponding to the three spectra of Figures 5(a–c), respectively. The accelerometer’s spectrogram captures the fundamental mode and a few harmonics.

The attached microphone has many harmonics as well as the fundamental mode. On the other hand, the detached microphone picks up a lot of noise from the environment. The results of this test case indicate the attached microphone can pick up the dominant modes of the accelerometer data. Moreover, many disturbances captured by the detached microphone can be excluded by the attached microphone.

**Figure 7.**
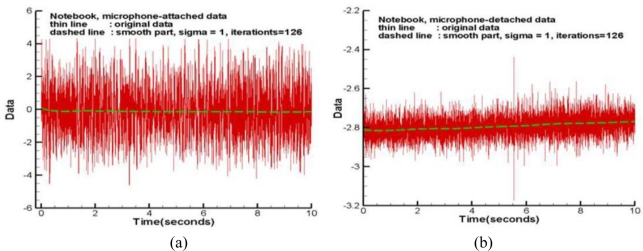
Raw data of a notebook computer: **(a)** microphone attached to the right board of touchpad. **(b)** microphone at 2 cm above the touchpad.

**Figure 8.**
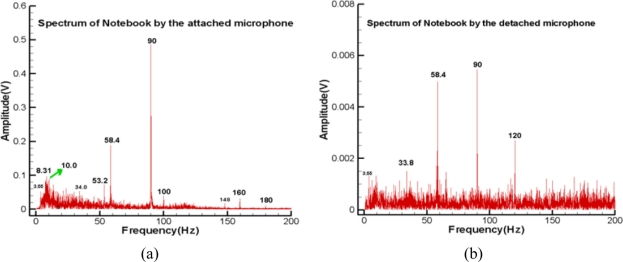
Spectrum of notebook computer: **(a)** microphone attached to the right board of touchpad. **(b)** microphone at 2 cm above the touchpad.

The last test examined the acoustic information from the fan and hard-disk of an ASUS X59SR series notebook computer. A microphone was attached to the right plate board of the touchpad by hand and the other was about 2 cm above the touchpad. During the data collection period, a long file was read from the hard-disk to the Random Access Memory (RAM) to examine the capability of these microphones. Figure 7(a,b) displays the raw data with a sampling rate of 1 kHz. The corresponding spectra of the high frequency parts are plotted in Figures 8(a,b), respectively. In the frequency range below 200 Hz, the detached microphone picked up four prominent modes, that is 33.8, 58.4, 90 and 120 Hz, respectively. Interestingly, the fundamental mode of the fan is 90 Hz, which is verified by a tachometer. The 120 Hz mode may be the harmonic of the power line frequency and the interaction between the 90Hz mode and its 1/2 sub-harmonic. Although much more information can be read from Figure 8(b), it is difficult to tell whether they are emitted from the fan and disk or somewhere else.

**Figure 9.**
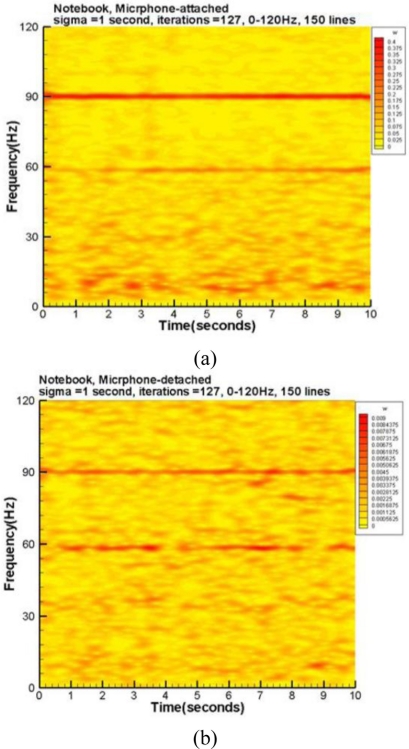
Spectrum registered by notebook computer: **(a)** microphone attached to the right board of touchpad. **(b)** microphone at 2 cm above the touchpad.

On the other hand, in addition to 34.0, 58.4 and 90 Hz modes, Figure 8(a) shows many prominent modes. The spectrum has 10 and 180 Hz modes, which are the 1/9 sub-harmonic and the first harmonic of the 90 Hz mode, respectively. The modes of 100 and 160 Hz are the combined modes related to the fundamental mode. Moreover, it has a band around 8.3 Hz. None of these modes can be clearly seen from Figure 8(b). The corresponding spectrograms are shown in Figure 9(a,b), respectively. Aside from the extremely low frequency region, these spectrograms repeat the same information as previously mentioned about the spectra. Note that Figure 9(a) has many short period segments in the band of 8–10Hz. This information reflects the short period motions of moving the read/write head of the hard disk. Figures 8(b) and 9(b) cannot fully capture the motions because their energies are too low to actuate the detached microphone. This practical test case indicates the attached microphone system can capture the small vibration signals and effectively exclude disturbances. The detached microphone cannot reproduce these signals because it cannot cutoff many signals emitted from other sources.

For clarity, the above discussions are summarized below:

1. The experimental setup confirms that, if the acoustic signal is strong enough, a commercial microphone can effectively capture acoustic signals in the 0.5–20 Hz range. Therefore, it can be used as a senor to pick up vibration information, provided the data acquisition system is properly arranged.
2. The vibration exciter test confirms the proposed microphone system can reflect the vibration information taken by the accelerometer together with some additional acoustic signals. In this test, the microphone is attached to a surface of the target. Most interested acoustic waves are confined to a small air chamber, ensuring sufficiently strong acoustic waves reach the microphone’s membrane.
3. A practical test case shows the proposed system effectively picks up small vibration information.

## 5. Conclusions

This study designs a specific experimental setup in which the acoustic wave reaching the electret condenser microphone’s actuating membrane is strong enough to be useful. In such a special environment, the microphone resolves acoustic signals in the 0.5–20 Hz range. Two experimental results also demonstrated the effectiveness that, by attaching the microphone to a plate surface of the target, a similar environment can be reproduced. Thus, much information in the low frequency region is captured by the proposed system. Since the system is simple, cheap, convenient, and precise, it can be considered potential vibration signal acquisition system.
